# New Roles for EVs, miRNA and lncRNA in Bovine Embryo Implantation

**DOI:** 10.3389/fvets.2022.944370

**Published:** 2022-07-15

**Authors:** Kazuhiko Imakawa, Yuta Matsuno, Hiroshi Fujiwara

**Affiliations:** ^1^Laboratory of Molecular Reproduction, Research Institute of Agriculture, Tokai University, Kumamoto, Japan; ^2^Department of Obstetrics and Gynecology, Kanazawa University Graduate School of Medical Science, Kanazawa, Japan

**Keywords:** extracellular vesicles (EVs), miRNA – microRNA, lncRNA – long non-coding RNA, implantation, ruminants

## Abstract

The sine qua non of new life is fertilization. However, approximately 50% of fertilized eggs/blastocysts in cattle and up to 75% of those from human assisted reproductive procedures fail during the first 3 to 4 weeks of pregnancy, including peri-implantation periods. In these periods, blastocyst hatching and implantation to the maternal endometrium proceeds, during which physiological events such as epithelial-mesenchymal transition (EMT) and trophoblast cell fusion occur. Quite recently, extracellular vesicles (EVs) with micro RNAs (miRNAs) and long non-coding RNAs (lncRNAs) have been found to play a pivotal role for the establishment of the proper uterine environment required for peri-implantation processes to proceed. New findings of EVs, miRNA, and lncRNAs will be described and discussed to elucidate their connections with conceptus implantation to the maternal endometrium.

## Introduction

Approximately 50% of fertilized eggs and/or blastocysts in cattle fail to continue their pregnancy during the first 3 to 4 weeks of pregnancy. During these periods, blastocysts and/or conceptuses (embryo plus extraembryonic membranes) undergo blastocyst hatching, conceptus elongation, conceptus attachment/adhesion to the maternal endometrium and subsequent placentation. It has been generally accepted that the communication between conceptus and endometrium *in utero* is crucial for the establishment of the proper uterine environment as well as maternal receptivity of the conceptus for pregnancy to proceed.

Communication between two cell types such as trophectoderm and uterine luminal epithelial cells is achieved through secretory molecules such as hormones and cytokines. These findings point to the importance of the analysis of cytokines and their receptor molecules using various methods including RNA-seq and proteomic analyses. In recent years, however, evidence has accumulated that extracellular vesicles [EVs, (1)] produced by the conceptus and endometrium play a role in communication between the two cell types. EVs, covered with a lipid bilayer, contain surface receptors and ligands, and cargo of proteins, lipids, metabolites, DNAs and RNAs from the originating cells. EVs with detergent-resistant membrane domains also contain relatively high concentrations of cholesterol, sphingomyelin, and ceramide, and these vesicles are stable in extracellular spaces (2).

In this review, recent findings of EVs, miRNA, and lncRNA functioning *in utero* during the early stages of bovine pregnancy will be presented and their roles in pregnancy success will be discussed.

## EV Cargoes and Their Effects/Function

It has been found that the cargoes of EVs differ according not only to cellular origin, but also to physiological and/or pathological conditions. In fact, EVs contain a variety of bioactive molecules including proteins, lipids, metabolites, DNAs, and RNAs (mRNA, miRNAs, and other RNA molecules) (3). In addition, there are numerous proteins and receptors, such as epidermal growth factor receptor (EGFR), bound onto the lipid layer of EVs. Through lipidomic analysis, EVs were found to contain cholesterol, sphingomyelin, ceramide, glycerophospholipids, phosphatidylcholine, and phosphatidylserine (1, 4–6). Proteomic analyses have also revealed that EVs contain different types of proteins such as heat shock proteins (HSP70 and HSP90), tetraspanins (CD9, CD63 and others), major histocompatibility complex class I and II (MHC class I and II), endosomal sorting complex proteins (Alix and Tsg101) and chaperons, most of which are often used for protein markers (1, 4, 5). Other cargoes of EVs are membrane trafficking proteins (Annexins, Flotillin and GTPases), cytoskeletal proteins (actin and tubulin), and numerous cytosolic proteins.

## Involvement of EVs in Pregnancy Establishment

### EVs and Conceptus Hatching and Elongation

It was previously thought that the early pre-implantation mammalian embryos are relatively autonomous, and that they control their own development. During the periods of peri-implantation, fertilized eggs/blastocysts go through numerous physiological changes including blastocyst migration, hatching, conceptus elongation, attachment/adhesion to the uterine epithelium, and placentation. These processes, particularly conceptus implantation and placentation, undoubtedly require not only their own gene functions but also maternal support (7). These two-way communications result in the generation of a proper uterine environment for both conceptus and endometrial developments. It was thought that various cytokines and their receptors play a role in conceptus-maternal communication (8). However, data has recently been accumulated that EVs, derived from both the conceptus trophectoderm and endometrium, play a role in bilateral communication between the conceptus and the endometrium (9–12).

The amount of EVs secreted from *in vitro*-cultured bovine embryos is correlated with embryo competence (13). In addition, the bovine embryo-derived EVs were found to improve the viability and growth of cloned embryos, as well as improve implantation rates and full-term calving rates (14). The correlation between EV secretion and *in vitro* embryo viability was also explored, and it was found that EV secretion during blastulation was indicative of viable bovine embryos. Further, EVs isolated from uterine flushing of pregnant and non-pregnancy female sheep (ewes) were tested for their ability to stimulate ovine trophectoderm (oTr1) cell proliferation. It was also found that EVs from pregnant ewes increased trophectoderm cell proliferation as well as the secretion of IFNT (15), the cytokine required for the prevention of corpus luteum demise in ruminants (16).

Non-invasive and elongated conceptuses initiate their attachment process to the uterine epithelium on day 16 in sheep and day 19 in cattle. Recent findings suggest that this is the time when the amounts of EVs *in utero* from both conceptus and uterine epithelium increase and play a role for conceptus growth, successful implantation and subsequent placentation (17–19).

### EVs and Conceptus Cell Fusion

In ruminant trophectodermal cells, the formation of bi- and multi-nucleate cells begins concurrent with initiation of conceptus attachment to the uterine epithelium. The cellular mechanisms associated with the bi- and multi-nucleate cells have not been well-characterized; at present, however, two theories on trophectoderm cell fusion exist: consecutive nuclear divisions without mitotic polyploidy (cytokinesis) (20) or the fusion of mononucleate cells (21). It has been believed for the last several decades that tri- or multi-nucleate cells found in the uterine endometrium of the ruminants result from the cell fusion between the bi- or multi-nucleate trophectoderm and the uterine epithelial cells (22). It was quite recently demonstrated that fused cells in the ovine species comprised all trophectodermal cells (23), agreeing with results recently published elsewhere (24).

It was demonstrated that endogenous retroviruses (ERVs), anciently infected and integrated into the genomes of vertebrates, are involved in the formation of trophoblast bi- and possibly multi-nucleate cells. In the bovine species, ERVs of syncytin-Rum1 (25) and BERV-K1/Fematorin-1 (26) have been found to exhibit cell fusogenic activity. In sheep, endogenous Jaagsiekte retroviruses (enJSRVs), of which the envelop genes are transcribed, are expressed in elongating ovine conceptuses after day 12 of pregnancy (21). Experiments with loss of enJSRVs function demonstrate that this treatment retards trophectoderm outgrowth and inhibits trophoblast bi-nucleate cell formation on day 16. Although these enJSRVs are packaged into endometrium-derived viral particles, another study on EVs provided evidence that between day 12 and 16 of gestation, shortly before the increase in EV exchange, the enJSRVs RNAs are packaged within the EV cargoes (27). These results support the idea that EVs could deliver ERVs, in addition to proteins, miRNA and others, from the endometrium to the conceptus.

### EVs and Conceptus Attachment/Adhesion to the Uterine Epithelium

Even in non-invasive placentation, elongated conceptus adhesion to the uterine epithelium is a typical prerequisite for placental formation in ruminant ungulates. It was found that bovine conceptus adhesion is mediated through a cell adhesion mediator, vascular adhesion molecule (VCAM-1) (28). Bovine uterine flushing (UF) containing EVs from the uteri of days 20 and 22 pregnancy has been shown to increase the expression of VCAM-1 in bovine endometrial epithelial cells (EECs) (29). Expression of VCAM-1 receptor, ITGA4, increased in day 22 bovine conceptuses (28). In addition, formation of fibrin at sites of conceptus adhesion has been demonstrated in the ewe (30). These observations strongly suggest that the bovine conceptus does adhere to the EECs on day 22, a couple days following the initiation of conceptus attachment to the uterine epithelium.

Epithelial-mesenchymal transition (EMT) is seen at the trophoblasts in the invasive mode of human and murine placentation. In 2012, however, Yamakoshi et al. found that most EMT-related factors are expressed in non-invasive bovine conceptuses on day 22 (31). The molecular mechanisms leading to conceptus EMT have been identified in the following two experiments. High expression of follistatin in bovine conceptuses on day 20 was down-regulated on day 22 and activin A, an EMT inducer, increased on the same day. Using bovine trophoblast CT-1 cells (32), a transcription factor OVOL2 was high on day 20. As OVOL2 expression decreases on day 22, EMT-related transcription factors, ZEB1 and SNAI2, along with mesenchymal cell markers, N-cadherin (CDH2) and vimentin (VIM), increase. Recently, Calle and coworkers executed an elegant experiment with the establishment of bovine trophoblast primary cells and endometrial mesenchymal cell lines (33). These authors demonstrated that interactions of both cell types through their secretomes (EVs and soluble proteins) lead to EMT in trophectodermal cells necessary for implantation and placentation (33). These investigators also found that after EMT, six proteins associated with the vascular endothelial growth factor (VEGF) pathway are increased (33), agreeing with the previous finding in which a micro-angiogenesis process related to uterine vascularization is necessary for implantation to proceed in the bovine species (34).

Proteomic analysis on human endometrial epithelial-derived EVs revealed that several members of the integrin family are found in these EVs, suggesting that these integrins play a role in docking these EVs to recipient cells and mediating trophoblast adhesion to endometrial cells through the interaction with appropriate ligands (35). In support of these observations, when EVs' miR-30d in the mouse endometrial fluid was transferred to murine embryos, they exhibited the up-regulation of cell adhesion molecules such as *Itgb3, Itga7*, and *Cdh5* (36). These results suggest that regardless of invasive or non-invasive mode of placentation, EVs play a significant role in conceptus attachment and/or adhesion to the endometrium. Together with secretomes, the significance of EVs in these processes is on the rise.

### EVs and Regulation of Immune Function

One of the crucial events that mammals must establish is maternal immunologic tolerance to the fetal allograft, which permits conceptus development *in utero* as well as the continuation of pregnancy. During the conceptus implantation period, EVs carry molecules likely to modulate the local endometrial (37) and possibly systemic immune systems (38). It was recently demonstrated that bovine EVs isolated from day 20 uterine flushing media (UFs) down-regulate the expression of immune-related genes in EECs (39). These investigators identified bta-miR-98 as a likely maternal immune system regulator. In dairy cows, Bta-miR-499, derived from placental exosomes, was found to regulate inflammation locally at the maternal-fetal annex through the inhibition of NK-kB signaling. Inhibition of bta-miR-499 results in deregulation of the inflammatory response at the maternal-fetal interface and fetal growth retardation (40). These results suggest that miRNA in intrauterine EVs play a role in the regulation of the local immune system to facilitate the continuation of pregnancy.

### EVs and Their Possible Regulation by Progesterone

The direct action of progesterone (P4), the essential requirement for pregnancy in mammals, on conceptus development has not yet been elucidated, although its action is likely to be mediated through endometrial gene expression (41, 42). It was found numerous times that uterine gene expression in ruminants is regulated by P4 and/or IFNT during the peri-implantation periods (7, 18, 43–46). One novel experiment has demonstrated the biological effect of P4 on the production of EVs: EVs found in endometrial luminal and glandular epithelia increase their numbers over two-fold when ovariectomized sheep are treated with P4 (47). In addition, the results from analyses of ovine endometrial miRNAs and EVs in the uterine lumen revealed that P4 regulates seven miRNAs, of which three miRNAs are down-regulated and four miRNAs are up-regulated (47). A similar experiment in humans found that P4 induces changes in EV production and their protein cargo of EECs, and these EVs could increase the adhesive capacity of human and mouse blastocysts (35). Although P4-regulated EV cargoes have not yet been fully characterized, these results clearly indicate that P4 increases the release of EVs from the endometrial epithelium, which can be detectable in the uterine lumen.

## Involvement of miRNA and lncRNA for Pregnancy Establishment

### miRNAs and Their Biogenesis

**Figure 1:**
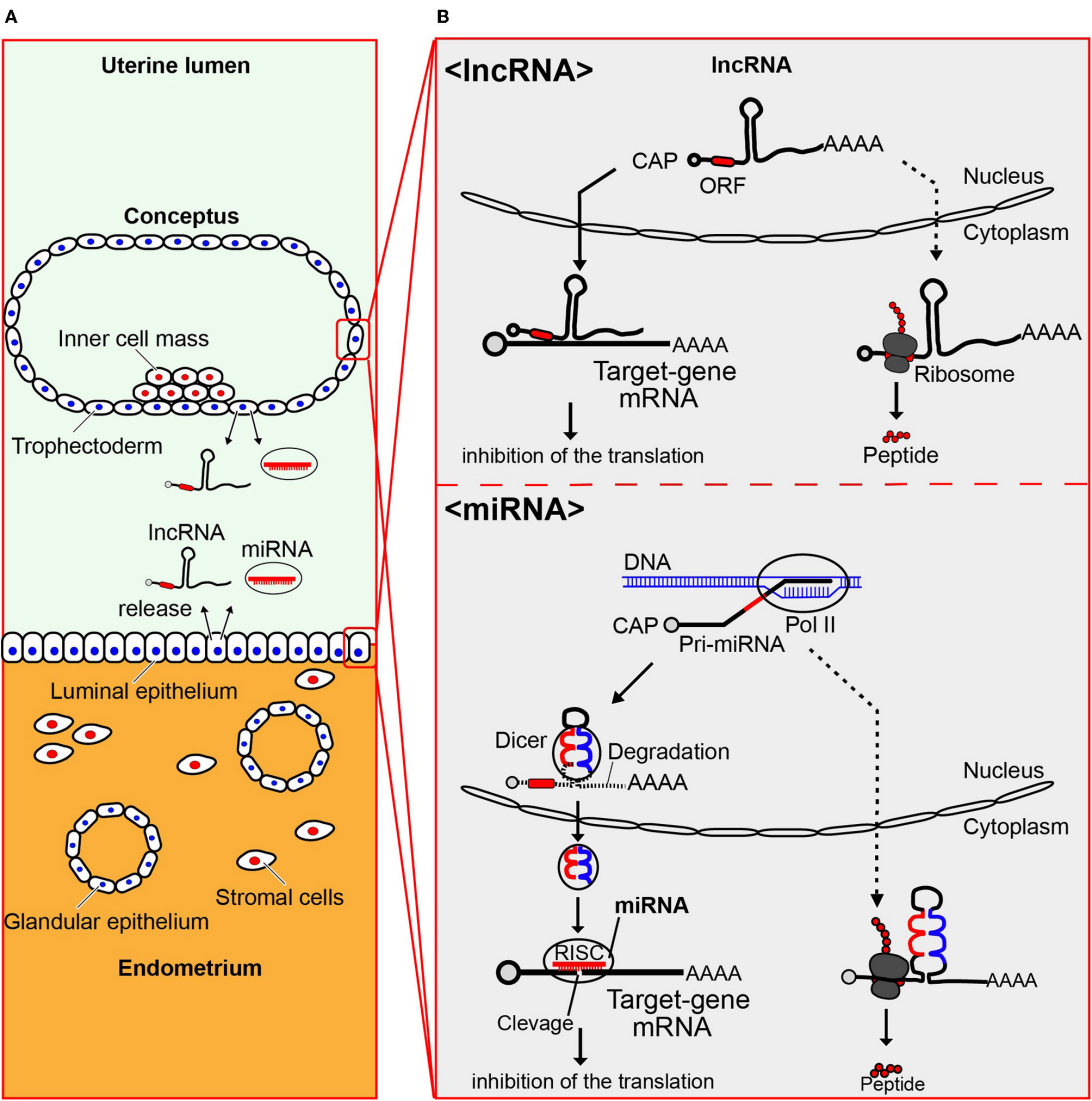
miRNA and lncRNA, produced by both conceptus and endometrium, are present in the uterus. **(A)** Uterine structure showing both maternal endometrium and conceptus (embryo plus extraembryonic membranes), from which miRNA and lncRNA are produced and miRNA in EVs and lncRNA are released into the uterine lumen. **(B)** Processing of lncRNA and miRNA is shown. Long non-coding RNAs (lncRNAs) are those with a length exceeding 200 nucleotides, some of which translate proteins/polypeptides (dotted-line arrow)._lncRNA can regulate the expression of target genes at the post-transcriptional level through the inhibition of their translation (solid-line arrow). Primary transcripts of miRNAs can be several kilobases in length, which are successfully cleaved by two RNase III enzymes, Drosha and Dicer, to produce approximately 70 nucleotide long precursor miRNA, and finally become 22–24 nucleotide long mature miRNAs (51) (solid-line arrow). The dotted-line arrow indicates that a part of those possibly produce polypeptides. When delivered into target cells, the miRNAs are likely to inhibit target mRNAs post-transcriptionally through the formation of RNA induced silencing complex (RISC), leading to changes in gene expression and cellular functions in distant cells.

It has been well-documented that miRNAs originate from large primary (pri) and precursor (pre) transcripts that undergo various processing steps till they reach mature and functional forms (48–50). Primary transcripts can be several kilobases in length but can be successfully cleaved by two RNase III enzymes, Drosha and Dicer, to produce approximately 70 nucleotide long precursor miRNA, and finally become 22–24 nucleotide long mature miRNAs (51). When delivered into target cells, the miRNAs are likely to inhibit target mRNAs post-transcriptionally through the formation of RNA induced silencing complex (RISC), leading to changes in gene expression and cellular functions in distant cells (Figure 1).

### lncRNA, and Functions of miRNAs and lncRNAs

Long non-coding RNAs (lncRNAs), defined as non-coding RNA sequences with a length exceeding 200 nucleotides, have emerged as important regulators in cellular functions such as translation of protein coding genes, signaling pathways, and epigenetic regulation (52, 53). These sequences have limited coding potential due to a lack of an open reading frame in the nucleotide segment. In cancer cells, lncRNAs have been extensively studied because they are potential regulators of many cellular mechanisms associated with cancer progression.

By binding to their target genes, miRNAs or lncRNAs regulate the expression of target genes at the post-transcriptional level, by inhibiting their translation. The miRNAs and lncRNAs should thus be treated with the same importance as those of target genes in the cellular development and differentiation processes. Most research related to miRNAs rests on the assumption that miRNAs exist intrinsically within many cell types. However, the mechanism by which miRNAs are sorted into EVs or retained in cells remains largely unknown.

### Emerging Role of miRNA in Conceptus Hatching and Elongation

Hatching from the zona-pellucida is a prerequisite for embryo implantation and is less likely to occur *in vitro* for reasons not yet characterized. In a recent experiment (54), miR-378a-3p, secreted by individually cultured bovine embryos, is shown to promote blastocyst hatching. The next event required for pregnancy establishment in ruminants is the proper conceptus elongation prior to the initiation of conceptus attachment to the uterine epithelium (55). Several molecules involved in the regulation of conceptus elongation have been identified through loss of function experiments: proline-rich 15 (PRR15), nuclear peroxisome proliferator activator receptors (PPARs), hydroxysteroid (11-beta) dehydrogenase 1 (HSD11B1), and LIN28A/B. Sheep conceptuses treated with the lentivirus expressing shRNA against PRR15 result in embryonic losses on day 15 (56). Sheep conceptuses treated with morpholino antisense oligonucleotide against PPARs or HSD11B1 also result in severely growth-retarded conceptuses or conceptus fragments on day 14 (57, 58). Furthermore, trophectoderm-specific knockdown of LIN28A/B in day 9 ovine blastocysts results in increased let-7 mRNA and reduced conceptus elongation on day 16 (59). It should be noted that while these three molecules play a role during the conceptus elongation *in utero*, sufficient conceptus elongation *in vitro* has not been demonstrated.

### Emerging Role of lncRNA in Conceptus Implantation

Expression of lncRNA in female reproductive tracts, particularly when expressed *in utero* during peri-implantation periods, has been characterized in pigs (60–63), goats (64), sheep (65), and mice (66). Quite recently, Matsuno et al. (67) analyzed the lncRNA profiles present *in utero* on days 15, 17, 19 and 21 of pregnancy in sheep. Among 8,808 lncRNAs identified, 3,423 lncRNAs were novel. Using gene ontology analysis, lncRNA target genes were enriched for cellular processes involved in the respiratory electron transport chain (RETC) (67). The results from these analyses suggest that in addition to secretomes, lncRNA is a potential new regulator in ovine conceptus development during peri-implantation periods.

## Recent Finding on miRNA/lncRNA Processing

Quite recently, Garcia-Martin and coworkers (68) have demonstrated sorting sequences of miRNA that determine their secretion in EVs (EXOmotifs) or cellular retention (CELLmotifs). In their observations, insertion or deletion of these CELLmotifs or EXOmotifs in a miRNA increases or decreases retention in the cell of production or secretion into EVs. In addition, two RNA-binding proteins, Alyref and Fus, are involved in the delivery of miRNA with the EXOmotifs, CGGGAG, resulting in increased miRNA export, which leads to enhanced inhibition of target genes in distant cells (68).

Another question is whether non-coding lncRNAs can translate polypeptides. If this were the case, what would be their functions? One such example would be the identification of pri-microRNA encoded polypeptide 133 (miPEP133) (Figure 1). This protein, encoded by the precursor of miR-34, functions as the tumor-suppressor in nasopharyngeal and ovarian cancer cells (69). These findings suggest that polypeptides translated from lncRNA could also be found in bovine uterine and/or conceptus cells.

It has been known that the testis expresses the largest number of tissue-specific lncRNAs (70, 71). A recent publication by Mise et al. (72) has demonstrated that those previously annotated as lncRNAs in the mouse can encode for two small and sperm-specific polypeptides, Kastor and Polluks. These polypeptides are found in the outer mitochondrial membrane and directly interact with the voltage-dependent anion channel (VDAC). Mice without both Kastor and Polluks expression exhibit severely impaired male fertility due to abnormal mitochondrial sheath formation. These authors conclude that cooperative function of these polypeptides in the regulation of VDAC3 may be essential for mitochondrial sheath formation in spermatozoa (72). Moreover, the relationship between lncRNA and spermatogenesis in association with the extracellular matrix and spermatogenesis (73) and in varicocele-induced spermatogenic dysfunction has also been demonstrated (74). These observations suggest that lncRNA, whether non-coding or coding small polypeptides, may become another molecule recognized as essential for vital aspects of the reproductive process.

## New Model of Pregnancy Establishment

It is generally accepted that IFNTs are confined *in utero*, and do not escape from the uterine lumen. Romero et al. (44) identified that the antiviral activity of IFN increases in the uterine vein during early pregnancy in sheep, of which activity on day 15 is blocked by anti-IFNT antibody. Accumulated data recently revealed that Interferon-Stimulated Gene, before (ISG) expression is up-regulated in peripheral blood mononuclear cells (PBMC) when IFNT is the only IFN present during this period (75, 76). Although further experimentation is required, uterine EVs, originating from both conceptuses and the uterine endometrium, could be taken up by uterine and conceptus cells, respectively. It is thus possible that those taken up by the endometrium enter the blood stream, which could up-regulate ISGs in PBMC.

Secretory products and their receptors *in utero* have been extensively studied for the last 4–5 decades. During the last decade, RNA-seq and iTRAQ analyses have been extensively used to identify key molecules that determine success or failure of early pregnancy. However, molecular, biochemical, and/or cellular mechanisms associated with early embryonic losses during the first 4 weeks of pregnancy have not been characterized. Recent data strongly suggest that in addition to those identified and characterized previously, miRNA in EVs and lncRNA must be evaluated within the context of the uterine environment required for pregnancy establishment and maintenance.

## Conclusion

Much research has been conducted to identify and elucidate target genes that encode for transcription factors, cytokines and their receptors. It has become apparent that EVs containing proteins, lipids, metabolites, DNAs, and RNAs (mRNA, miRNAs, and other RNA molecules) play a role in many biological processes, including reproduction. Evidence is mounting that miRNA and lncRNA are deeply involved in the regulation of multiple biological processes required for successful conceptus implantation to the maternal endometrium and placentation. In addition to paracrine actions *in utero*, it is possible that these molecules encapsulated in EVs could escape from the original cells or tissues and enter circulation. When these EVs reach target cells, they undoubtedly change the paradigm of pregnancy-associated gene regulation as well as phenotypic changes in distant cells or tissues.

## Author Contributions

KI had ideas of writing review manuscript on miRNA and lncRNA, which are becoming important molecules in the outcome of implantation in ruminants. YM and HF agreed with the concept and wrote parts of the manuscript. Figure was generated by YM. All authors contributed to the article and approved the submitted version.

## Funding

This work was supported by Livestock Promotional Funds of Japan Racing Association (JRA) (KI) and partly by KAKENHI Grants-in-Aid for Scientific Research (16H02584 to KI) from the Japan Society for the Promotion of Science.

## Conflict of Interest

The authors declare that the research was conducted in the absence of any commercial or financial relationships that could be construed as a potential conflict of interest.

## Publisher's Note

All claims expressed in this article are solely those of the authors and do not necessarily represent those of their affiliated organizations, or those of the publisher, the editors and the reviewers. Any product that may be evaluated in this article, or claim that may be made by its manufacturer, is not guaranteed or endorsed by the publisher.
